# Case of Typhoid Fever

**Published:** 1877-08-20

**Authors:** Z. B Herndon

**Affiliations:** Richmond, Va.


### CASE OF TYPHOID FEVER.

By Z. B HERNDON, M.D., Richmond, Va.

On the 9th September, 1874, I was called to see an old gentleman, aged 73, and, after attending him some days, was satisfied that he had typhoid fever.

He was put upon nutritious diet, consisting of Liebig's essence of beef, essence of beef "home-made," chicken jelly, milk punch, egg-nog, etc. Other articles were also tried, but of everything he soon tired, except the milk punch, which was his constant diet for fourteen months, during which time all efforts to induce him to take additional food were fruitless. A few trials of any article soon brought disgust.

The daily quantity of milk varied from one quart to three pints made into a stiff punch with good whiskey, and was taken at intervals of two hours.

On several occasions death seemed imminent. At such times I sat by him and gave, in addition to the quantity of whiskey punch mentioned, brandy and carb. ammonia freely through the night.

During the whole attack there was but little opportunity for giving medicine. Turpentine every three or four hours, with Dover's powder at night, seemed to be beneficial. Different tonics, vegetable and mineral acids, were used during convalescence. Before recovery was fully established the lower limbs were attacked with a miserable eczema, which for a long time seemed to defy everything in the way of general and local treatment. Nothing afforded so much comfort as water in which was soaked the bran of wheat.

After rising from bed the process of reform was commenced. While my patient was a man of strictly temperate habits and great moral strength, it was interesting to observe how he seemed to be aware of every drop of spirit that was withheld, although the diminution was by the teaspoonful.

The system became tolerant of an enormous quantity of spirits without ever showing any symptoms of excess.

Time and perseverence established the old habit of strict temperance, and now the subject of this report weighs twenty-five pounds more than at any period of his life.

It will be observed that no new remedies are here suggested, but I hope interest will be found in these facts:

1. The age of the patient.
2. Duration of the disease.
3 Tolerance of one article of diet.
4. Persevering stimulation when death *seemed* to be master of the situation.
5. The patient is as strictly temperate as before the attack.

connected with some degree of hysteria.

After treating a few cases, unsatisfactorily to myself, (and them, too, I suppose,) I began the use of fl. ext. gelsem. gtt. x., with ammon. b omid gr. x. to xv., morning and evening; and have yet to prescribe these remedies for the case that was not materially improved or entirely cured in a compara tively short time.

Dysmenorrhea, or other complications, require their appropriate treatment. Many cases of so-called ovaritis, (sub-acute or chronic, (are of a neuralgic character, and benefitted by hypodermic injections of morphia and ergot, (fl. ext. in small doses.

PHLEGMASIA DOLENS.

During the past year three well-marked cases of this disease occurred in my practice.

One followed an abortion, at tenth week, in which the hemorrhage had been alarming, and, contrary to the usual termination described in "the books," suppurated; was lanced in the popliteal spece, and again a few days later, at inner side of ankle, more than a quart of pus being evacuated from each incision.

In the second case, five weeks had elapsed from date of the confinement, when disease appeared, and in this case hemorrhage had been profuse after delivery.

In third case, the disease occurred three weeks after delivery, in case of entire placenta prævia in primipara, in which the operation of turning the child had been performed while the patient was under chloroform, and in which there had been excessive loss of blood from repeated floodings during the last two weeks of gestation, as well as during labor, before sufficient relaxation to admit of operative iuterference. These cases seem to uphold the theory that excessive loss of blood is predisposing, if not remotely, an exciting cause of this disease.

The treatment in all the cases was turpentine stupes, and warm water dressing,—opium sufficient to subdue pain and calm nervous irritation, good nourishing diet, with tonics and stimulants as symptoms and condition of patien required. Nature is probably our b st aid in these cases.

PUERPERAL MANIA.

A lady, aged thirty-two, mother of four children, was delivered of her fifth child, 28th February, 1876, and two days later became furiously maniacal, and unmanageable, having homicidal tendencies. Sixteen months previ usly, on birth of her fourth child, she suffered a similar attack, but not so severe in character, for which she had been treated with chloral and potass. bromid., with excellent results.

In last attack, these remedies failed to be of much use, and tr. lupulin, in fl.dr.ij doses, with fl. ext. gelsem. gitt. viij. was given every two, three or four hours, as needed to produce calmative effect. In a couple of days these medi–cines seemed to loose their beneficial effect. Patient was now freely purged by calomel and fl. ext. senna; the nucha freely blistered, and hypodermic injections of $\frac{1}{4}$ gr. m rphia with tinct. aconit rad. gtt. j. were used morning and evening, with the effect of controlling the cerebral excitement, though producing rather an unpleasant degree of narcotism the first few doses, which, however, passed by safely, and patient made a good recovery, having been deranged about ten days.

The probability is that another attack may be recorded in my case book ere long.

This patient's father was insane for a short time, a few years ago, but seems perfectly recovered.

