# Correction

**Published:** 1877-12-20

**Authors:** 


Correction.—Our attention has been drawn to an error in the form of our August number, by which the article of Dr. Z. B. Herndon, on typhoid fever, seems to run into another article. His paper really closes at the bottom of page 204. It seems that oversights will occasionally occur, despite the most watchful attention.

